# Supplementary material for: Morphological and functional variability in central and subcentral motor cortex of the human brain
Source: Brain Struct Funct. 2020 Dec 23;226(1):263–79. doi: 10.1007/s00429-020-02180-w (PMC7817568; doi:10.1007/s00429-020-02180-w)
Supplement: Supplementary file 1 — Supplementary file1 (DOCX 1091 kb) [file 429_2020_2180_MOESM1_ESM.docx]

**Supplementary Material**

*Anatomical Characterization of Sulcal Segments based on Morphological Types*


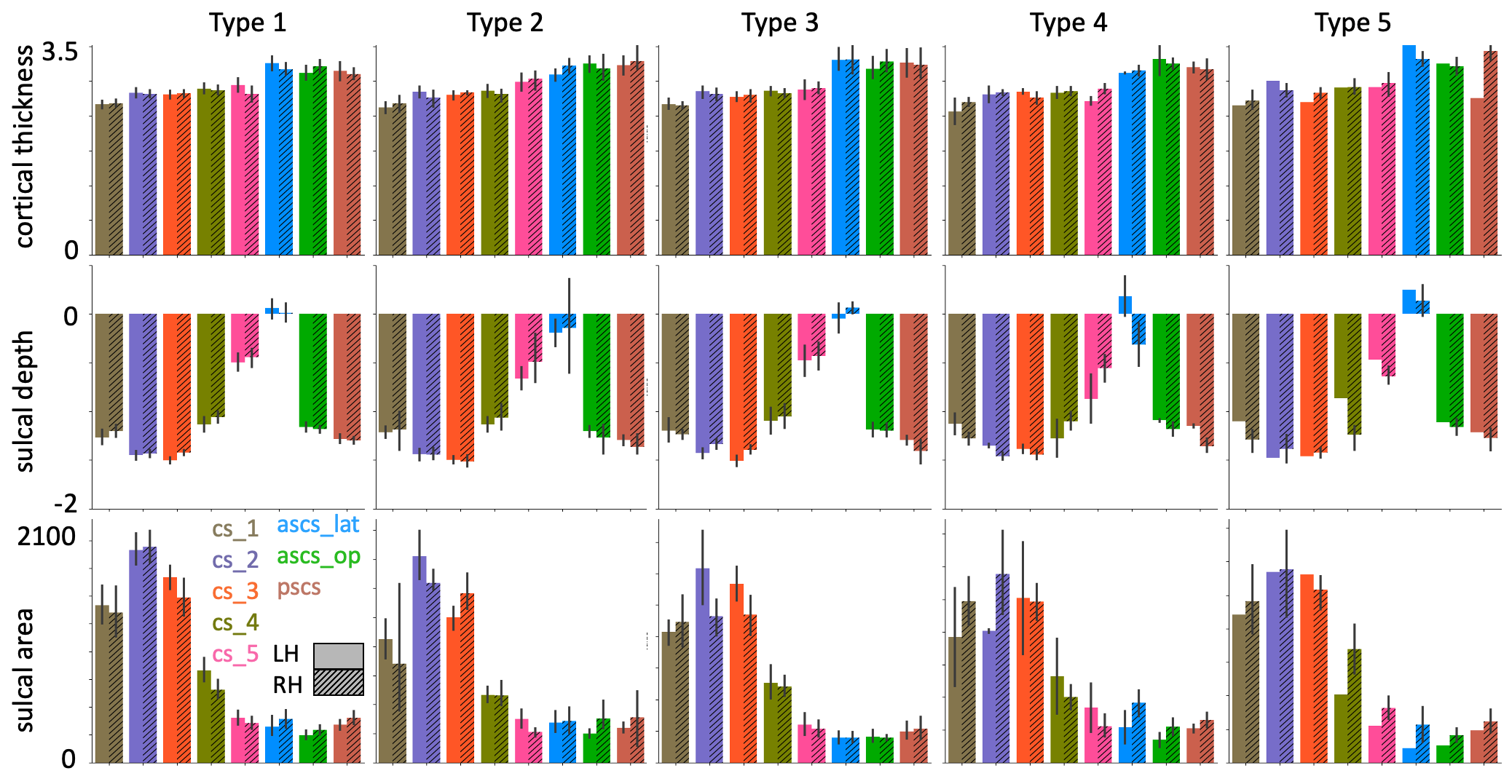


**Fig. 7** Anatomical measures for sulcal segments in different morphological types. Morphological types refer to the patterns in the subcentral region as shown in Fig. 2. For more details on the figure description see Fig. 4. Sample sizes underlying each type and hemisphere are provided in Table 1

*Regions-of-Interest to derive Maxima during Task Activation*

### The definition of regions of interest is described, in detail, in Eichert et al. (2020a) and is repeated here in a modified version. The central sulcus ROI used for the hand, lip and tongue was defined using FreeSurfer’s automatic volumetric labeling based on the Destrieux Atlas.

For the larynx, we identified two activation maxima in separate ROIs: One for the dorsal and one for the ventral larynx representation. The dorsal larynx ROI was a portion of the same central sulcus ROI used above from *z*-coordinates in MNI space of 50 - 30. The limits were determined empirically, so that the dorsal larynx ROI did not capture the ventral larynx representation or an unrelated supra-dorsal activation in the trunk area, which was observed in some individuals (Foerster 1931).

The ventral larynx representation lay outside the central sulcus and was located ventrally in the subcentral part of cortex. Because of the high inter-individual morphological variability in this region, the ventral larynx ROI was derived manually based on individual anatomy in surface space. A liberal surface ROI was drawn on each individual’s mid-thickness surface covering the ventral part of the central sulcus and adjacent gyri. Anteriorly, the ROI was delineated by the inferior portion of the precentral sulcus and, posteriorly, the ROI spanned the postcentral gyrus. If present, the lateral portion of the anterior sulcus in the subcentral gyrus was included within the ROI. The dorsal limit of the ROI was defined by a horizontal plane across the gyrus at the level of the usual location of the posterior ramus of the inferior precentral sulcus. The ventral larynx surface ROI was converted into a volumetric ROI covering the underlying cortical ribbon using wb_command. We checked that the ventral larynx ROI did not overlap with subjacent auditory cortex in the temporal lobe or inferior frontal cortex.

In some participants, the main contrast for vocalization in the syllable production task had additional activity related to articulation of the tongue. To remove this additional activity, we transformed the coordinates for each individual’s maximal voxel from the tongue contrast (from the basic localizer task) to the functional space of the syllable production task (task 1) using rigid-body transformation and then derived a spherical ROI (7 voxels in diameter) around it. This sphere was used to mask the *z*-statistic image of the main contrast for vocalization prior to localizing the maxima for laryngeal activity in the dorsal and ventral ROIs described above.

*Animation of one Individual’s Sulcal Segments*

**Online Resource 1** The animation shows the 3D rendering of one individual’s sulcal segments in native volume space, i.e. the same participant as in Fig. 1 using the same color legend.
